# *HMGA1* gene expression level in cancer tissue and blood samples of non-small cell lung cancer (NSCLC) patients: preliminary report

**DOI:** 10.1007/s00438-022-01936-9

**Published:** 2022-08-10

**Authors:** Lias Saed, Ewa Balcerczak, Mariusz Łochowski, Ewa Olechnowicz, Aleksandra Sałagacka-Kubiak

**Affiliations:** 1 Department of Pharmaceutical Biochemistry and Molecular Diagnostics, Medical University of Lodz, Lodz, Poland; 2 Department of Thoracic Surgery, Memorial Copernicus Hospital, Medical University of Lodz, Lodz, Poland

**Keywords:** HMGA1, Gene expression, Lung cancer, Prognosis, Liquid biopsy

## Abstract

The study aimed to assess the *HMGA1* gene expression level in NSCLC patients and to evaluate its association with selected clinicopathological features and overall survival of patients. The expression of the *HMGA1,* coding non-histone transcription regulator HMGA1, was previously proved to correlate with the ability of cancer cells to metastasize the advancement of the disease. The prognostic value of the *HMGA1* expression level was demonstrated in some neoplasms, e.g., pancreatic, gastric, endometrial, hepatocellular cancer, but the knowledge about its role in non-small cell lung cancer (NSCLC) is still limited. Thus, the *HMGA1* expression level was evaluated by real-time PCR method in postoperative tumor tissue and blood samples collected at the time of diagnosis, 100 days and 1 year after surgery from 47 NSCLC patients. Mean *HMGA1* expression level in blood decreased systematically from the time of cancer diagnosis to 1 year after surgery. The blood *HMGA1* expression level 1 year after surgery was associated with the tobacco smoking status of patients (*p*= 0.0230). Patients with high blood *HMGA1* expression levels measured 100 days after surgery tend to have worse overall survival than those with low expression levels (*p*= 0.1197). Tumor *HMGA1* expression level was associated with neither features nor the overall survival of NSCLC patients. Moreover, no correlation between *HMGA1* expression level measured in tumor tissue and blood samples was stated. Blood *HMGA1* mRNA level could be a promising factor in the prognostication of non-small cell lung cancer patients.

## Introduction

Despite advances in the understanding of risk factors, pathogenesis, and treatment methods for lung cancer, it remains one of the most prevalent and also the most deadly neoplasm worldwide. According to data published by The Global Cancer Observatory, lung cancer accounted for 11.4% of all malignant neoplasms diagnosed in the world in 2020. Almost one in five cancer deaths in 2020 in the world was caused by lung cancer (W.H.O 2020). As prognosis and treatment outcomes of lung cancer patients are strongly influenced by the clinical advancement of the disease at the time of diagnosis, new prognostic and predictive factors are still being sought.

Non-small cell lung cancer (NSCLC) is the most common lung cancer type accounting for 85% of its cases. The development of NSCLC is driven by genetic mutation, the most important of which are those in epidermal growth factor receptor (EGFR) and anaplastic lymphoma kinase (ALK) genes (Gridelli et al. 2015). In-depth analyses of genomes and signaling pathways revealed the NSCLCs is a group of distinct diseases with genetic and cellular heterogeneity, which determine the optimal management strategy, including surgery, radiochemotherapy, immunotherapy and targeted approaches with tyrosine kinase inhibitors or anti-angiogenic monoclonal antibodies. The complexity of somatic alterations in NSCLCs includes transcription factors, splicing factors and epigenome modifiers, genes involved in cellular immunity (Chen et al. 2014).

High-mobility group AT-hook 1 (HMGA1, previously HMG-I/HMG-Y) is a chromatin architectural factor that binds to the minor groove of AT-rich DNA. It influences chromatin structure enabling transcription factors to assembly and control the expression of various downstream genes. In this way, HMGA1 is involved in crucial cellular processes like differentiation, apoptosis, DNA damage repair, cell cycle regulation, cellular senescence, but also in neoplastic transformation. It is encoded by the *HMGA1* gene which produces two protein isoforms HMGA1a and HMGA1b identical in sequence except for the deletion of the 11 amino acid stretch in the HMGA1b protein between the first and second DNA-binding domains (Sumter et al. 2016).

In addition to its presence in the nucleus of HMGA1, where it acts as a transcription regulator of different signaling pathways, it has been discovered that it may also be secreted and plays its oncogenic role outside the cell. Méndez et al. (2018) showed the blocking of extracellular HMGA1 decreased metastatic burden in a xenograft model of triple-negative breast cancer (TNBC). Moreover, in TNBC patients, an extracellular location of HMGA1 was connected with the incidence of metastasis. They also demonstrated that extracellular HMGA1 is a ligand for the Advanced glycosylation end product-specific receptor (RAGE), inducing pERK signaling and increasing migration and invasion of cancer cells.

The expression level of *HMGA* genes is high during embryonic development, but low or undetectable in completely differentiated, non-dividing cells of a mature organism, which implies the involvement of the products of expression of these genes in the regulation of cellular proliferation and embryonic growth (Reeves 2001; Reeves & Beckerbauer 2001; Wisniewski & Schwanbeck 2000). Contrary to normal somatic cells, the levels of *HMGA1* products are often increased in tumor-transformed cells and correlate with the advancement of the disease or the ability to metastasize. Association between the overexpression of full-length HMGA proteins and tumor progression was found in colorectal, prostate, breast, and thyroid cancer (Wisniewski & Schwanbeck 2000).

The expression of both the *HMGA1* gene and the HMGA1 protein was proved to be up-regulated in many different lung cancer cell lines compared with normal human lung bronchial epithelium cells (Hillion et al. 2009). Also, most primary lung tumors showed higher expression of the *HMGA1* gene than in the normal lung tissue (Hillion et al. 2009; Sarhadi et al. 2006). Zhang et al. (2019) showed that high HMGA1 expression in the NSCLC tissue is connected with a higher TNM stage. NSCLC patients with higher staining of the HMGA1 in cancer tissue had worse OS than those the lower staining. Based on publicly available cancer data, *HMGA1* was shown to be overexpressed in both SCLC and NSCLC, with higher expression compared to both the adjacent non-malignant lung tissues and non-tumor lung tissues of healthy individuals. Elevated *HMGA1* expression was connected with some clinicopathological features like sex, age, and TNM stage. The high *HMGA1* expression level was connected with shorter overall and first progression survival time among lung adenocarcinoma patients, but not lung squamous cell carcinoma patients (Saed et al. 2022). However, the exact role of *HMGA1* in lung cancer pathogenesis is not fully understood.

Some studies provide evidence that *HMGA1* is responsible for carcinogenic dysregulation of crucial gene pathways or miRNAs in many tumor types, including lung cancer (Pallante et al. 2015). RNA profiling of lung epithelial cells expressing a mutant allele of *PIK3* revealed HMGA1 is a part of transcription factor network connected with aberrant PIK3/AKT signaling in lung cancer, and NSCLC-derived cultured cells with activated AKT presented higher *HMGA1* expression levels than the cells with low AKT activation (Scrima et al. 2012). The bioinformatic analysis identifies the *HMGA1* to be one of the top 10 genes in the miRNA-gene regulatory network in NSCLC (Zhou et al. 2020). Zhang et al. (2011) showed that in NSCLC cells *HMGA1* directly enhances the transcriptional activity of miR-222 and in consequence, it upregulates pAKT signaling. Induction of IL-24, a novel tumor suppressor cytokine in the H1299 lung cancer cell line, was associated with markedly reduced *HMGA1* expression level, miR22-3p and -5p levels with a concomitant decrease in pAKT expression (Panneerselvam et al. 2016). Hillion et al. (2009) demonstrated that *HMGA1* can act as an oncogene that drives cell transformation in undifferentiated, large-cell lung cancer phenotype. This process is mediated by *HMGA1*-dependent upregulation of the matrix metalloproteinase-2 gene (*MMP-2*) which promotes migration and invasion of H1299 large-cell carcinoma cells. Building a HMGA1-centered protein–protein interaction network (Saed et al. 2022) revealed that the protein could interact with proteins involved in cellular senescence and cell cycle control (TP53, RB1, RPS6KB1, and CDK1), transcription regulation (EP400 and HMGA2), chromatin assembly and remodeling (LMNB1), and cholesterol and isoprene biosynthesis (HMGCR and INSIG1).

Additionally, HMGA1 could also contribute to the poor outcome of lung cancer treatment using both conventional and targeted drugs. Recently it was demonstrated that the protein participates in the forkhead box protein M1-high-mobility group AT-hook 1-G6PD (FOXM1-HMGA1-G6PD) transcriptional regulatory pathway which activated enhanced the cisplatin resistance of NSCLC cells (Zhang et al. 2019). Some evidence was shown that the phosphorylation level of HMGA1 protein contributes to EGFR tyrosine kinase inhibitor resistance by affecting EGFR downstream signaling. Knockdown of *HMGA1* expression in human lung adenocarcinoma cell line reinforced gefitinib efficiency by reactivation of EGFR or PDGF downstream signaling (Wang et al. 2017).

Blocking the HMGA1 function could have therapeutic implications**.** Decreased proliferation and enhanced apoptosis were observed in human thyroid anaplastic carcinoma cell lines after the suppression of HMGI(Y) protein synthesis by an HMGI(Y) antisense adenoviral vector (Scala et al. 2000). Similarly, a reduction in cell viability and increased sensitivity to gemcitabine was observed in five different pancreatic and liver cancer cell lines after infection with replication-defective engineered adenovirus containing the HMGA1 decoy hyper binding sites (Hassan et al. 2018). siRNA-mediated knockdown HMGA1 in combination with IL-24 reduced markedly AKT expression and substantially reduced migration and invasion of cultured lung cancer cells (Panneerselvam et al. 2016).

Considering the limited knowledge about the role of the *HMGA1* in lung cancer transformation as well as putative prognostic, predictive, and therapeutic value of the gene, we aimed to appraise the importance of the *HMGA1* expression level in tissue and blood samples of NSCLC patients. The presented study provides some evidence that tissue *HMGA1* expression is markedly up-regulated during carcinogenesis in the lung. Moreover, blood *HMGA1* mRNA level could decrease in time after tumor resection and could be connected with the survival of patients.

## Materials and methods

### Investigated group

The investigated group comprised 46 NSCLC patients (8 females and 38 males) diagnosed and treated at the N. Copernicus Regional Specialist Hospital in Lodz, Poland. The mean age at the time of diagnosis was 66.9 years (min. 54 years, max. 82 years). All patients underwent surgical resection. In 16 cases, adjuvant chemotherapy was included after the surgery (carboplatin + gemcitabine 2 or cisplatin + etoposide 2 or etoposide 2 or cisplatin + vinorelbine 9). Between 2016 and 2018, tissue sections and peripheral blood samples were collected from the patients: 40 blood samples—at time of diagnosis of cancer; 39 blood samples—100 days after the surgery; 24 blood samples—1 year after the surgery, and 46 frozen tissue sections taken intraoperatively.

### RNA isolation

RNA from blood and tissue sections were isolated according to “Blood Mini” and “Total RNA Mini” protocol, respectively (*A&A Biotechnology*, Poland). The purity and concentration of RNA samples were assessed spectrophotometrically. The concentration of extracted RNA ranged from 5.2 to 80.0 ng/μl. RNA samples were stored at− 76 °C until the analysis.

### Reverse transcription

A total RNA was transcribed into complementary DNA (cDNA) following the High-Capacity cDNA Reverse Transcription Kit protocol (*Applied Biosystems,* USA). The final concentration of RNA in the reaction mixture was 0.02 μg/μl. Obtained cDNA was stored at− 20 °C until analysis. To check cDNA synthesis efficacy, PCR for *GAPDH* gene was conducted. In all samples, the presence of PCR product for the *GAPDH* (133 bp) was stated.

### Real-time polymerase chain reaction (real-time PCR)

Quantification assessment of expression of both the investigated gene and the reference gene was performed by real-time PCR using the Rotor-Gene 6000 (*Corbet Research, Germany*). The reaction mixture for both genes consisted of 10 μl 2 × Bimake™ SYBR Green qPCR Master Mix, 0.3 μl of each primer (*HMGA* gene: F 5′-CAACACCTAAGAGACCTCG -3′, R 5′-TCCTCTTCCTCCTTCTCC-3′; *GAPDH* gene F.

5′-ACAGTTCCCATGTAGACC-3′, R 5′-TTGAGCACAGGGTACTTTA -3′), 0.5 μl of cDNA and distilled water up to 20 μl final volume. The reactions for *HMGA* and *GAPDH* were carried out in separate tubes. For confirmation of reaction specificity, melting curve analysis after each reaction set was carried out. The temperature profile of real-time PCR was as follow: hold 95 °C/10 min, cycling (40x)—95 °C/10 min, 58 °C/15 s, 72 °C/20 s, melting 72 °C–95 °C. Samples were tested in triplicates and the mean of obtained Ct values for both genes was calculated. In each experiment, negative control (distilled water instead of cDNA) also in triplicates, was included. Additionally, relative standard curves for both investigated and reference genes were plotted. To this basis, the efficiency of the reactions was determined: 95.18% for the *HMGA1* gene, 101.26% for the *GAPDH* gene. The relative expression level of the investigated gene was calculated using a method developed by Pfaffl (2001) and analyzed after the decimal logarithmic transformation.

### Statistical analysis

Statistical analysis of data generated in the research was performed using Dell Statistica (data analysis software system), version 13 (Dell Inc., 2016, software.dell.com). The conformity of continuous variables with normal distribution was checked using the Shapiro–Wilk test. To determine the significance of differences in gene expression according to clinical data, analysis of variance with repeated values, paired-sample t-test, Student t-test, Pearson and Spearman correlation was used. Survival analysis was conducted applying Kaplan–Meier plots and log-rank test. A *p-*value < 0.05 was assumed as significant in all tests conducted.

## Results

### The expression level of HMGA1 gene in tumor tissue and blood samples

**Fig. 1:**
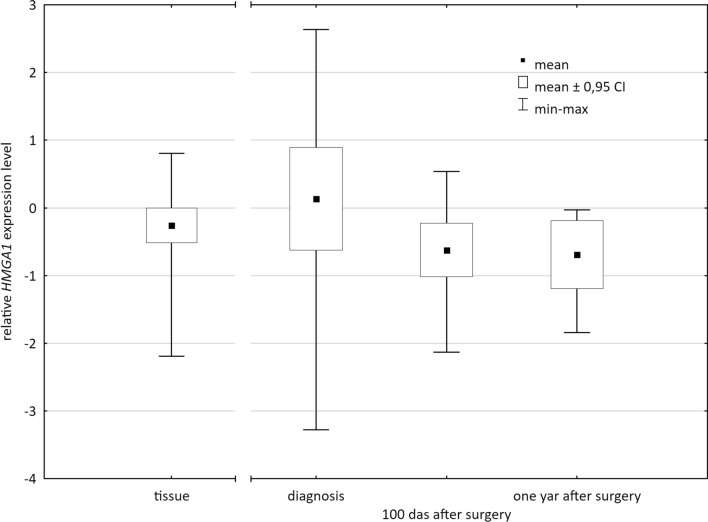
Relative *HMGA1* expression level in cancer tissue and blood samples at three time points

*The HMGA1* expression level was successfully determined in 28 blood samples collected at the time of diagnosis of cancer, 21 blood samples collected 100 days after the surgery, and 11 blood samples collected 1 year after the surgery. The mean blood expression level decrease in time (Fig. 1.). The blood expression level at the time of cancer diagnosis was higher than the level measured 100 days after with marginal statistical significance (*p* = 0.0502). The level at the time of diagnosis was also visibly higher than the level measured 1 year after surgery but the difference was not significant (*p* = 0.1838). There was no significant difference in the level between samples collected 100 days and 1 year after surgery (*p* = 0.8350).

The *HMGA1* expression level was also determined in 46 frozen tissue sections taken intraoperatively. The levels of expression measured in cancer tissue and blood samples taken at the time of diagnosis did not differ significantly (*p* = 0.8290). However, the expression level in tissue was significantly higher than the level determined in blood samples collected after treatment—both 100 days and 1 year after surgery (*p* = 0.0429 and *p* = 0.0182, respectively, Fig. 1.).

### Association between the expression level of HMGA1 gene and selected clinicopathological features

**Fig. 2:**
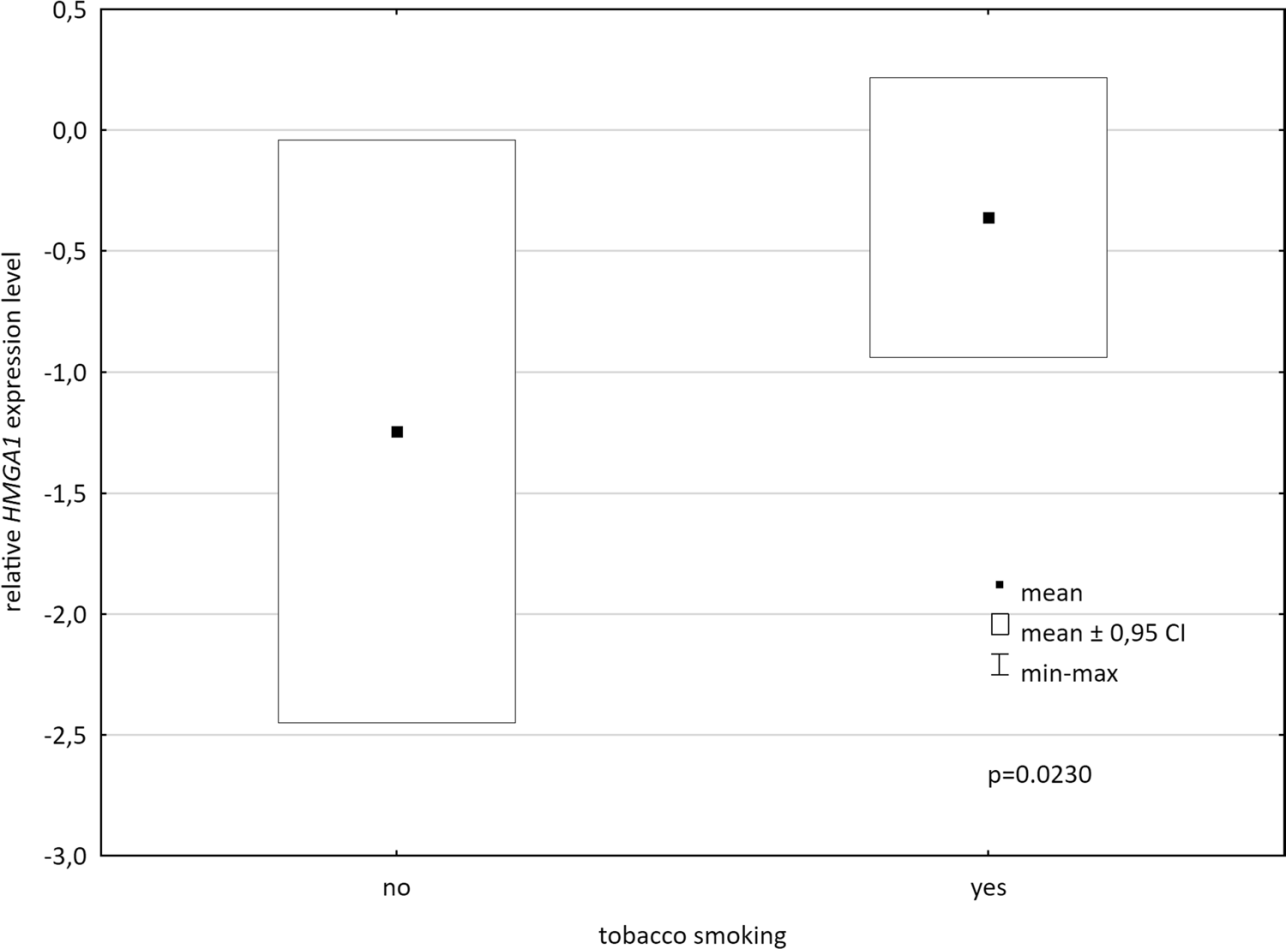
Relative *HMGA1* expression level measured 1 year after surgery concerning tobacco smoking status

Next, the connection between the *HMGA1* expression level in tissue and blood samples and selected clinicopathological features was soughed (Table 1.). The level was compared between subgroups of patients divided according to gender, tobacco smoking status, histological type of cancer, TNM stage, and grade of malignancy. The *HMGA1* expression level measured 1 year after surgery was significantly higher in smokers than in non-smokers (*p* = 0.0230) (Fig. 2). *P*-values for the analyzed association are summarized in Table 1.

**Table 1:** *P*-values for the association between relative *HMGA1* expression level and clinicopathological features

Feature	Relative *HMGA1* expression level			
	Cancer tissue (*n* = 46)	Peripheral blood		
		At the time of diagnosis (*n* = 28)	100 days after surgery (*n* = 21)	1 year after surgery (*n* = 11)
Gender (men vs woman)^a^	0.3261 (38/8)	0.7452 (21/7)	0.6038 (16/5)	0.4882 (9/2)
Age^b^	0.7912	0.3915	0.2206	0.2466
Tobacco smoking (smokers vs non-smokers)^a^	0.2283 (29/17)	0.6048 (15/13)	0.2812 (12/9)	0.0230 (7/4)
Histological type (adenocarcinoma vs squamous cell carcinoma)^a^	0.5626 (26/20)	0.4077 (14/14)	0.4687 (11/10)	0.6383 (6/5)
TMN stage (IA1 and/or IA2 and/or IB vs IIA and/or IIB and/or IIIA)^a^	0.4339 (22/24)	0.9230 (13/15)	0.5674 (10/11)	0.8057 (6/5)
Grade of malignancy (G1 and/or G2 vs G3)^a^	0.7157 (34/12)	0.8433 (21/7)	0.9085 (16/5)	0.3688 (9/2)

^a^Student's *t*-test

^b^Spearman's correlation rank

### The expression level of HMGA1 gene and survival of NSCLC patients

**Fig. 3:**
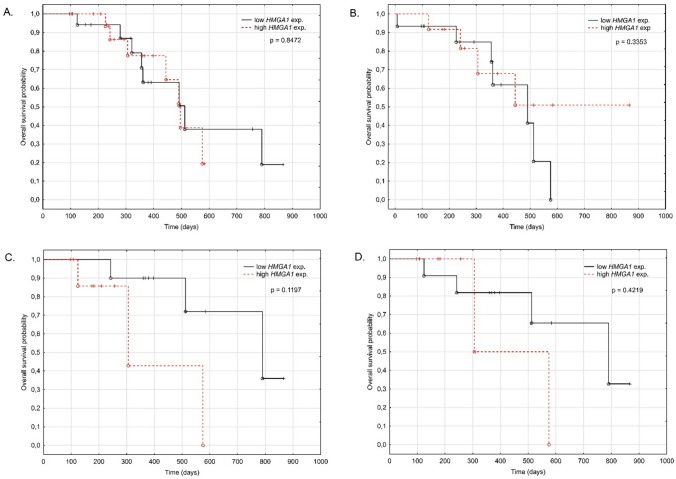
Overall survival probability according to *HMGA1* expression level measured in cancer tissue (**A**) and blood samples collected at the time of diagnosis (**B**), 100 days after surgery (**C**), and 1 year after surgery (**D**)

Lastly, the relationship between the overall survival time of patients and the level of the *HMGA1* expression in NSCLC tissue was analyzed. For this purpose, the study cohort was split into two subgroups with the level of *HMGA1* expression above (low expression *n* = 24) and below (high expression *n* = 22) the mean level of the entire study group. We did not state any association between the probability of survival of the patients and the *HMGA1* expression level (*p* = 0.8472, log-rank test; Fig. 3A.).

Next, an analogous analysis for the level of *HMGA1* expression in blood samples for each time point studied (diagnosis, 100 days after surgery, and 1 year after surgery) was done. At the time of diagnosis, the level of *HMGA1* expression did not differentiate patients in terms of survival time (low expression *n* = 18, high expression *n* = 16, *p* = 0.3353; log-rank test; Fig. 3B.). However, when the expression level measured 100 days after surgery was analyzed, a clear trend towards reduced over survival time in the high-expression patients was stated (low expression *n* = 10, high expression *n* = 11; *p* = 0.1197; log-rank test; Fig. 3C). The same but less pronounced tendency was observed when overall survival time concerning the *HMGA1* expression level measured 1 year after surgery was inspected. However, no statistically significant difference in the OS between the high and low expression level subgroups was revealed (low expression *n* = 5, high expression *n* = 6, *p* = 0.4219; log-rank test; Fig. 3D.).

## Discussion

Overexpression of the *HMGA1* gene, as well as increased levels of the protein encoded by this gene, was confirmed in many different lung cancer cell lines compared to normal epithelium bronchitis (Hillion et al. 2009; Ma et al. 2019). Also, most of the primary lung tumors studied showed higher *HMGA1* gene expression than normal lung tissue (Barh et al. 2013; Hillion et al. 2009; Ma et al. 2019). The overexpression of the *HMGA1* gene and its protein in lung cancer tissue, as well as their involvement in key signaling pathways in the development of the tumor, justified the search for their prognostic significance in lung cancer. However, only a few research reports about the importance of the *HMGA1* gene and HMGA1 protein expression as a prognostic factor in lung cancer are published to date. Zhang et al. (2015) showed that high HMGA1 protein expression in neoplastic tissue of NSCLC was associated with the size of the neoplastic tumor, the presence of lymph node and distant metastases, and with higher advancement according to the TNM classification, and these observations were confirmed by the results obtained by Zhang et al. (2019). Contrarily, in our study, we did not find the association between *HMGA1* gene expression level determined in NSCLC tissue and the TNM stage.

Previously, it was observed that a number of positively staining cells for HMGA1 by immunohistochemistry was lower in metaplasia and higher in dysplasia and carcinoma in situ of the bronchial epithelium (Sarhadi et al. 2006) which could suggest that *HMGA1* expression increases during carcinogenesis progress. The connection between HMGA1 protein expression in NSCLC tissue and poor histological differentiation of tumors was concordantly reported by Zhang et al. (2015) and Lin and Peng (2016) but not by Zhang et al. (2019). We did not find the association between *HMGA1* gene expression level determined in NSCLC tissue and grade of malignancy in our study group.

Kettunen et al. (2004) showed that the *HMGA1* gene is commonly up-regulated in both adenocarcinoma and squamous cell carcinoma of NSCLC, and Sarhadi et al. (2006) detected HMGA1 protein in a high proportion of lung cancer tumors irrespective of the histological type. No significant difference in the *HMGA1* gene expression level between cancer tissues of different histological types was stated in our study. Similarly, no major difference between NSCLC histological subtypes in *HMGA1* gene or protein expression was observed by Zhang et al. (2014) and Zhang et al. (2019) but HMGA1 protein was detected more often in the squamous cell carcinoma subtype compared to adenocarcinoma by Lin and Peng (2016).

Previously published research did not confirm the presence of an association between the HMGA1 protein expression in lung cancer tissue and age, gender, or smoking status of enrolled patients (Zhang et al. 2015, 2019; Lin and Peng 2016) expect the research of Lin and Peng (2016) who observed the tissue HMGA1 expression more frequently in males than females. Similarly, in our study, the level of *HMGA1* expression measured in cancer tissue was not connected with age, gender, or smoking status.

As *HMGA1* gene or HMGA1 protein expression level was associated with such features as the stage of TNM or histological differentiation of neoplastic cells, it can be presumed that the level of its expression will be related to the survival time of patients. Indeed, Sarhadi et al. (2006) showed a correlation between the nuclear expression of the HMGA1 protein and the shorter survival time of patients with lung adenocarcinoma (but not SSC), although this expression was not related to the proliferation or the apoptotic index of cancer cells. Moreover, nuclear staining of HMGA1 protein remained a significant factor influencing survival probability, even after taking into account the confounding variables in multivariate analysis. Similarly, in the research conducted by Zhang et al. (2015), high HMGA1 protein expression turned out to be an independent negative prognostic factor and was associated with a shorter survival time of NSCLC patients. However, not all studies conducted so far have confirmed the association between the amount of HMGA1 protein in neoplastic tissue and the survival time of lung cancer patients. Such a correlation was not noted by Lin and Peng (2016), although in their study the presence of the HMGA1 protein in NSCLC tissue samples was associated with poor histological differentiation of the tumor. According to the Kaplan–Meier Plotter (Ma et al. 2019), high expression of the *HMGA1* gene was connected with a shorter survival time of patients with NSCLC. However, we also did not state any association between *HMGA1* mRNA level in NSCLC tissue and overall survival time in our study group. It could be speculated that the effect of the *HMGA1* on the progression of NSCLC is probably dependent on other factors. For instance**,** in a study published by Zhang et al. (2019) the increased amount of HMGA1 protein found in NSCLC tissue was connected with shorter overall survival compared to patients with a low amount of HMGA1. However, the amount of HMGA1 protein in cancer tissue correlated with the amount of FOXM1 and G6PD, with which HMGA1 forms a common pathway for transcriptional regulation and which were similarly associated with TNM stage and overall survival (Zhang et al. 2019).

In the presented research, we screened for the first time the expression of the *HMGA1* gene in the peripheral blood samples of NSCLC patients and its changes in time. To date, some studies have investigated the *HMGA2* gene, another member of the high-mobility group family, to identify molecular diagnostic and prognostic markers in cancer using minimally invasive processes. Sezer et al. (2000) detected the *HMGA2* expression in the blood samples of metastatic breast cancer patients but not healthy donors nor non-metastatic patients. Moreover, the presence of *HMGA2* expression correlated with a worse prognosis. Later, Langelotz et al. (2003) stated that the presence of the *HMGA2* expression detected in the peripheral blood of metastatic breast cancer patients is connected with disease-specific survival and remains an independent prognostic for overall survival. On this basis, the gene was proposed as a potential marker for the early detection of circulating tumor cells in peripheral blood, but further research did not confirm these findings (Fabjani et al. 2005). *HMGA2* expression detectable in peripheral blood of CML patients correlated significantly with WBC count which indicates that the overexpression is connected with the undifferentiated phenotype of leukemic cells accumulation during the progression of chronic state to blast crisis (Meyer et al. 2007). Galdiero et al. (2015) proposed the circulating HMGA2 specific mRNA as a tool for early detection of epithelial ovarian cancer as it was found exclusively in the plasma of cancer patients but not healthy donors. The level of circulating mRNA was significantly higher than in healthy volunteers and substantially associated with tumor location, nerve infiltration, vascular invasion, MSI status and serum CA199 level (Sahengbieke et al. 2018). These promising findings encouraged our research on NSCLC cancer.

To our best knowledge, there were no data available to date on the expression of *HMGA1* in the peripheral blood of cancer patients except this published by Barth et al. (2013). Using the microRNA expression profile, in silico analysis based on reverse-transcriptomics, and interactome analysis, they selected seven transcription factors that could be biomarkers in the diagnosis of lung cancer. One of them was the *HMGA1*, which was significantly overexpressed in neoplastic tissue and the blood of both in lung squamous cell carcinoma and lung adenocarcinoma patients in the validation experiment. The *HMGA1* gene also belonged to a panel of selected transcription factor genes, the expression of which allowed for the differentiation of small cell and non-small cell carcinoma. Increased expression of the *HMGA1* gene was found in the blood of a limited cohort of NSCLC patients, compared to healthy subjects (Barh et al. 2013). We found that the *HMGA1* expression in the blood tends to decrease after surgical removal of the NSCLC tumor that could suggest tumor cells are the substantial source of *HMGA1* expression measured in the blood. It is believed that circulating tumor cells are shredded into the circulation by tumors in the early stages of cancer and are responsible for the development of latent metastases (Grzybowska and Fabisiewicz 2017). However, according to the Human Protein Atlas (Uhlen et al. 2017) the *HMGA1* expression was detected in all blood cell types, so the type of cells being the source of measured *HMGA1* transcript level should be specified in the future. We did not state a significant connection between the clinical advancement of the cancer disease and blood *HMGA1* expression level. Despite that, a clear tendency towards shorter over survival time in patients with high *HMGA1* expression levels measured 100 days after surgery was noted.

We also stated that the *HMGA1* expression level measured 1 year after surgery was significantly higher in smokers. Previously, it was found that the overall mortality of ever-smokers is higher than that of never-smokers in NSCLC patients, and current smoking is an independent risk factor for a poorer prognosis (Lee et al. 2014). Recently, some connection between the *HMGA1* expression and tobacco smoking status in lung adenocarcinoma (LUAD) was shown by Jung et al. (2021). When comparing TCGA data for the smoker LUAD and normal lung tissue, they found *HMGA1* as one of the six genes up-regulated and simultaneously inversely correlated with DNA methylation level in LUAD. This finding was further validated in clinical specimens of the smoker and never-smoker LUAD patients, revealing *HMGA1* displays borderline differences in CpG methylation between comparing cohorts. As it is widely known that cigarette smoke affects DNA methylation, and thus is a critical factor in the development of lung cancer (Zong et al. 2019; Dammann et al. 2005), it may, at least in part, explain the observed differences in blood *HMAG1* expression level associated with smoking status.

In conclusion, we examined, for the first time, the expression level of the *HMGA1* gene parallelly in tissue and blood samples of NSCLC patients. We screened the changes of *HMGA1* expression levels in the blood and noted the expression tends to decrease after surgical removal of the tumor over time. Our findings suggest the blood *HMGA1* expression level could be connected to the progression of the disease. These valuable results encourage us to broaden the study by the HMGA1 protein analysis in the future. Since detection of the expression might be useful for the selection of treatment schedule as well as monitoring of therapy response, it warrants further investigation in the field.
